# Inhibition of Ebola Virus by a Molecularly Engineered Banana Lectin

**DOI:** 10.1371/journal.pntd.0007595

**Published:** 2019-07-29

**Authors:** Evelyn M. Covés-Datson, Julie Dyall, Lisa Evans DeWald, Steven R. King, Derek Dube, Maureen Legendre, Elizabeth Nelson, Kelly C. Drews, Robin Gross, Dawn M. Gerhardt, Lisa Torzewski, Elena Postnikova, Janie Y. Liang, Bhupal Ban, Jagathpala Shetty, Lisa E. Hensley, Peter B. Jahrling, Gene G. Olinger, Judith M. White, David M. Markovitz

**Affiliations:** 1 Medical Scientist Training Program, University of Michigan, Ann Arbor, Michigan, United States of America; 2 Department of Microbiology & Immunology, University of Michigan, Ann Arbor, Michigan, United States of America; 3 Integrated Research Facility, Division of Clinical Research, National Institute of Allergy and Infectious Diseases, National Institutes of Health, Frederick, Maryland, United States of America; 4 Division of Infectious Diseases, Department of Internal Medicine, University of Michigan, Ann Arbor, Michigan, United States of America; 5 Department of Cell Biology, University of Virginia, Charlottesville, Virginia, United States of America; 6 Department of Pathology, University of Virginia, Charlottesville, Virginia, United States of America; 7 Antibody Engineering and Technology Core, University of Virginia, Charlottesville, Virginia, United States of America; 8 Emerging Viral Pathogens Section, National Institute of Allergy and Infectious Diseases, National Institutes of Health, Frederick, Maryland, United States of America; 9 Department of Microbiology, University of Virginia, Charlottesville, Virginia, United States of America; 10 Cellular and Molecular Biology Program, University of Michigan, Ann Arbor, Michigan, United States of America; 11 Graduate Program in Immunology, University of Michigan, Ann Arbor, Michigan, United States of America; 12 Cancer Biology Program, University of Michigan, Ann Arbor, Michigan, United States of America; Institute of Tropical Medicine, BELGIUM

## Abstract

Ebolaviruses cause an often rapidly fatal syndrome known as Ebola virus disease (EVD), with average case fatality rates of ~50%. There is no licensed vaccine or treatment for EVD, underscoring the urgent need to develop new anti-ebolavirus agents, especially in the face of an ongoing outbreak in the Democratic Republic of the Congo and the largest ever outbreak in Western Africa in 2013–2016. Lectins have been investigated as potential antiviral agents as they bind glycans present on viral surface glycoproteins, but clinical use of them has been slowed by concerns regarding their mitogenicity, i.e. ability to cause immune cell proliferation. We previously engineered a banana lectin (BanLec), a carbohydrate-binding protein, such that it retained antiviral activity but lost mitogenicity by mutating a single amino acid, yielding H84T BanLec (H84T). H84T shows activity against viruses containing high-mannose *N*-glycans, including influenza A and B, HIV-1 and -2, and hepatitis C virus. Since ebolavirus surface glycoproteins also contain many high-mannose *N*-glycans, we assessed whether H84T could inhibit ebolavirus replication. H84T inhibited Ebola virus (EBOV) replication in cell cultures. In cells, H84T inhibited both virus-like particle (VLP) entry and transcription/replication of the EBOV mini-genome at high micromolar concentrations, while inhibiting infection by transcription- and replication-competent VLPs, which measures the full viral life cycle, in the low micromolar range. H84T did not inhibit assembly, budding, or release of VLPs. These findings suggest that H84T may exert its anti-ebolavirus effect(s) by blocking both entry and transcription/replication. In a mouse model, H84T partially (maximally, ~50–80%) protected mice from an otherwise lethal mouse-adapted EBOV infection. Interestingly, a single dose of H84T pre-exposure to EBOV protected ~80% of mice. Thus, H84T shows promise as a new anti-ebolavirus agent with potential to be used in combination with vaccination or other agents in a prophylactic or therapeutic regimen.

## Author summary

There are no approved vaccines or treatments to combat infections with ebolaviruses, which cause Ebola virus disease (EVD), an often rapidly fatal disease characterized by fever and bleeding that results in death in up to ~90% of cases. Ebolaviruses are among the most pathogenic viruses that cause human disease and represent a threat to global public health. Outbreaks of EVD occur periodically in African countries and can be exported elsewhere, with recent outbreaks including one ongoing in the Democratic Republic of the Congo and the largest ever in Western Africa in 2013–2016. There is therefore a great need to develop new vaccines and treatments that target ebolaviruses. We examined whether a lectin (carbohydrate-binding protein), predicted to bind to carbohydrates present on the surface of many viruses and thereby interfere with infection, could block ebolavirus infection and be used for prevention and/or treatment of EVD. We found that the protein blocked ebolavirus infection in cell cultures and, moreover, protected a significant proportion of ebolavirus-infected mice from death, even when administered only once before exposure to virus as a preventive. The protein hence shows promise as a potential agent to prevent and/or treat EVD.

## Introduction

Filoviruses, which include the five species of *Ebolavirus*, are among the direst of all human pathogenic viruses, causing severe disease in humans and nonhuman primates that is often fulminant and rapidly fatal. Ebola virus disease (EVD) has a case fatality rate of 25–90%, with an average case fatality rate of 50% [1]. Since the discovery of ebolaviruses in 1976, there have been sporadic outbreaks of EVD in African countries, the longest and deadliest of which was the 2013–2016 Western African Ebola epidemic in Guinea, Liberia, and Sierra Leone, with approximately 28,000 cases and 11,000 deaths [2]. The latest outbreaks of EVD occurred from May to July of 2018 in the Democratic Republic of the Congo, with another unlinked outbreak ongoing in the same country as of August 2018, underscoring the unabating potential of ebolaviruses to re-emerge. Due to their high fatality rates and potential to be weaponized, filoviruses are considered category A priority pathogens and bioterrorism agents by the National Institute of Allergy and Infectious Diseases and the Centers for Disease Control and Prevention, respectively, and represent a serious threat to global health.

Unfortunately, there is currently no licensed vaccine or treatment for this deadly disease despite fervent efforts to develop preventive and therapeutic agents. Though progress has been made in developing anti-ebolavirus agents, challenges still remain. A number of ebolavirus vaccines have demonstrated safety and at least some immunogenicity in people [3,4], but the only ebolavirus vaccination trial to date to obtain clinical efficacy data was the Ebola Ça Suffit! trial, which took place during the 2013–2016 epidemic. This study employed ring vaccination of people epidemiologically linked to patients with EVD. Clinical trial subjects were vaccinated with the recombinant vesicular stomatitis virus vaccine (rVSV-ZEBOV), a live attenuated vaccine expressing the EBOV glycoprotein (GP) in a VSV vector [5]. Although the trial reported 100% efficacy in ring-vaccinated individuals and rVSV-ZEBOV is being used successfully in the current (August 2018 to manuscript submission) outbreak, there are challenges with its administration and it is not yet clear whether the vaccine will provide long-term protection [6–8]. Three experimental agents demonstrating at least some pre-clinical efficacy were used in the 2013–2016 outbreak [9], including ZMapp, a cocktail of three monoclonal antibodies against EBOV [10]; favipiravir, a viral RNA polymerase inhibitor [11]; and in two patients the nucleotide analog prodrug remdesivir [12,13], but neither these nor other antiviral therapeutic agents have yet been proven effective in clinical trials. Both ZMapp and vaccines require a cold chain, which would complicate distribution of these agents in the event of an outbreak given the poor infrastructure of many affected areas, and ZMapp is costly and requires administration by skilled health care workers. Given the paucity of licensed anti-ebolavirus vaccines and therapeutics, there is a critical need to develop new and potent agents for EVD prevention and treatment.

Lectins, or carbohydrate-binding proteins, have been viewed as candidate antiviral agents since many recognize glycans on the surfaces of viruses that are not commonly present on most normal human cells [14,15]. However, clinical use of lectins has been slowed by their mitogenicity, or ability to cause the proliferation of cells, particularly immune cells, that could potentially result in an unwanted immune reaction. We recently rationally engineered a lectin derived from bananas, banana lectin (BanLec), to retain broad-spectrum antiviral activity while removing mitogenicity with a single amino acid substitution, resulting in H84T BanLec (H84T) [16]. H84T binds to high-mannose *N*-glycans and has antiviral activity against a number of high-mannose-expressing viruses, including HIV-1 and -2, hepatitis C virus, and influenza A and B virus, among others. Ebolaviruses express, in addition to other glycans, a high level of high-mannose *N-*glycans on their surface glycoprotein (GP) molecules [17–19], envelope proteins important for viral entry [20]. Considering that H84T has demonstrated antiviral activity against other viruses containing high-mannose and that high-dose treatment with mannose binding lectin, another lectin with specificity for mannose, has proven efficacious in limiting EVD in vivo [21], in the present study we sought to characterize whether H84T has anti-ebolavirus activity and whether H84T could in turn protect against EVD in vivo. We demonstrate that H84T inhibits ebolavirus activity in vitro, protects mice from ebolavirus infection, and may exert its anti-ebolavirus effect by blocking both the entry and transcription/replication phases of the viral life cycle.

## Methods

### Cells and reagents

HEK293T/17 cells (ATCC CRL-11268) were obtained from the University of Virginia Tissue Culture Facility and were maintained in Dulbecco’s Modified Eagle Medium (DMEM) containing 1% sodium pyruvate, 1% L-glutamine, 1% antibiotic-antimycotic (all Gibco Life Technologies), and 10% supplemented calf serum (Hyclone). Vero E6 (ATCC CRL-1586, Manassas, VA), HeLa (ATCC CCL-2), and Huh 7 (human hepatocellular carcinoma) cells were maintained following recommended protocols.

**Fig 1:**
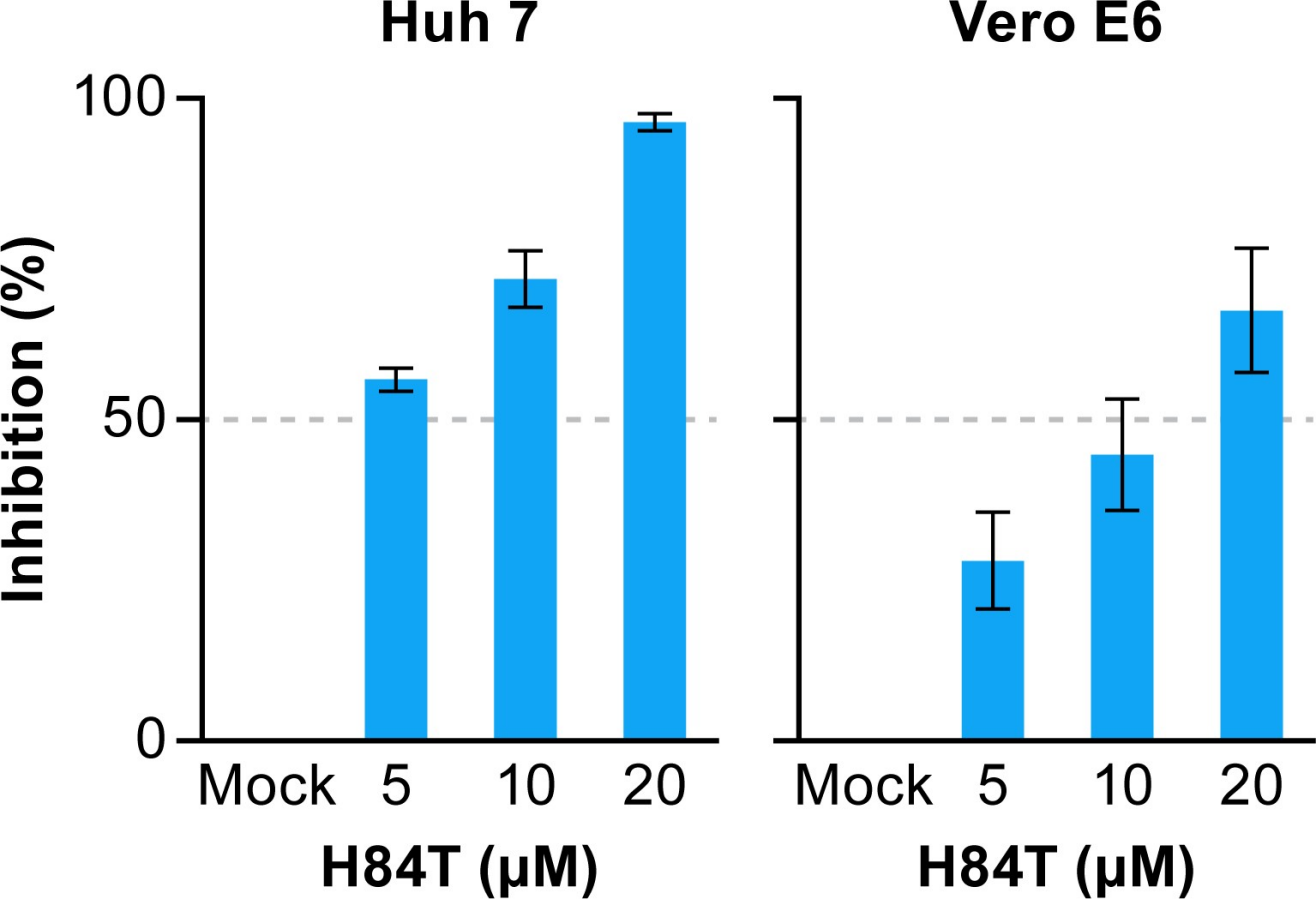
H84T inhibits Ebola virus infection in cell cultures. Huh 7 or Vero E6 cells were pretreated for 1 h with the indicated concentrations of H84T. Cells were infected with EBOV/Mak at an MOI of 2.5. After 48 h, cells were fixed and stained with an antibody to the EBOV VP40 protein followed by secondary staining with an Alexa 594-labeled antibody. The experiment was run on duplicate plates with triplicate wells per dose (mean ± SD; n = 3). One representative graph (from one of the two plates) is shown. Percent inhibition of infection was calculated as described in the Methods section. Abbreviations: EBOV/Mak, Ebola virus (Makona); MOI, multiplicity of infection; SD, standard deviation; VP40, viral protein 40.

**Fig 2:**
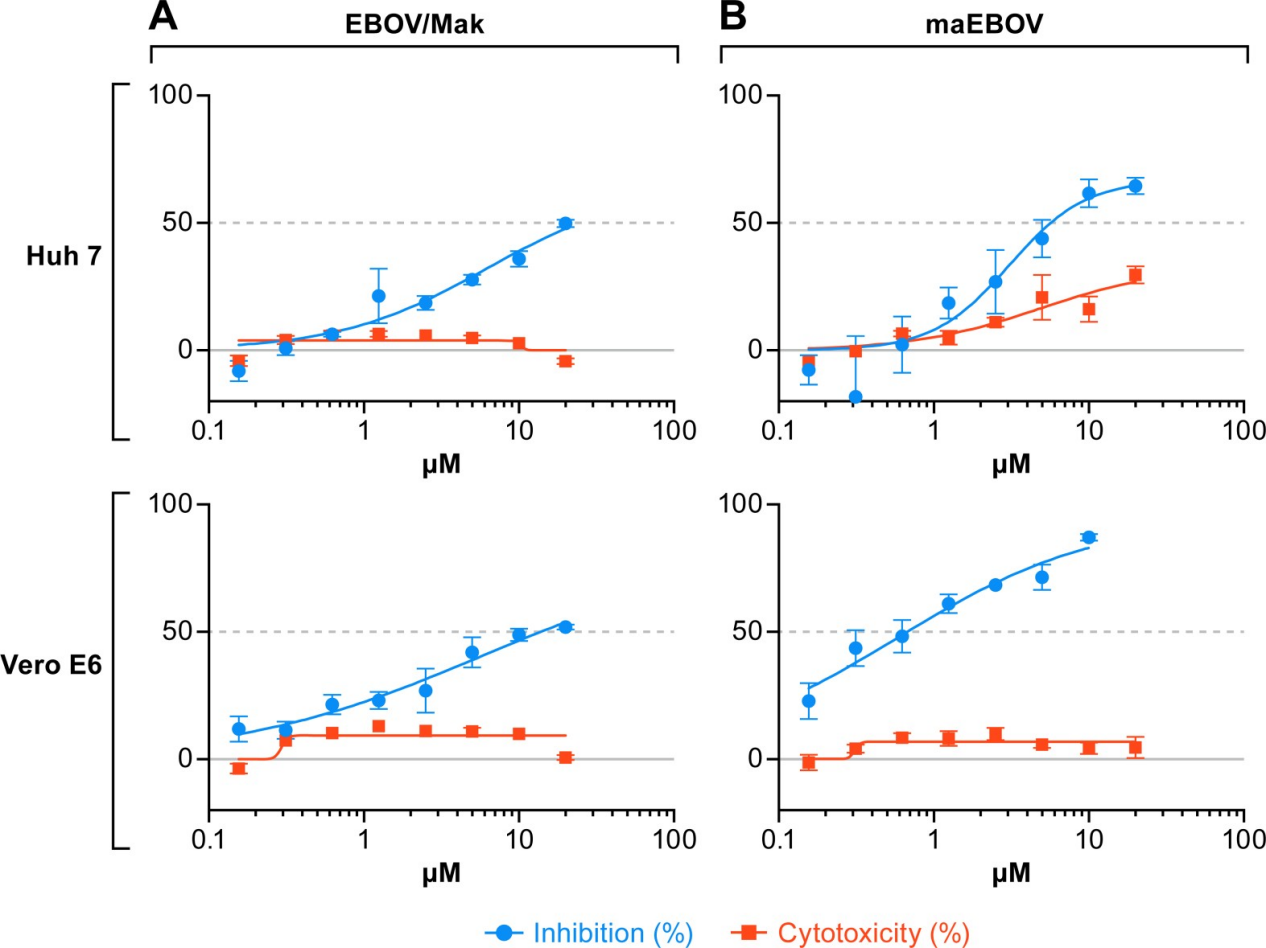
H84T inhibits human- and mouse-adapted Ebola virus replication in tissue culture. Huh 7 or Vero E6 cells were pretreated for 1 h with the indicated concentrations of H84T. Cells were subsequently infected with (A) EBOV/Mak at an MOI of 0.5 or (B) ma-EBOV at an MOI of 0.25 for 48 h. Cells were fixed and stained with an antibody to the EBOV VP40 protein followed by secondary staining with an HRP-labeled antibody. Antiviral activity of H84T, shown in blue, and cytotoxicity in uninfected cells, shown in red, were calculated as described in the Methods section. The experiment depicted in (A) was run twice for each cell line, and each experiment comprised duplicate plates with triplicate wells per dose (mean ± SD; n = 3). One representative graph from the two independent experiments (total of 4 plates) is shown. In (B), one experiment was run on a single plate with triplicate wells per dose (mean ± SD; n = 3). Average cytotoxicities (with 20 μM H84T) were 4.2% and 3.0% for Huh 7 and Vero cells, respectively (from n = 5 plates each). Abbreviations: EBOV/Mak, Ebola virus (Makona); HRP, horseradish peroxidase; ma-EBOV, mouse-adapted Ebola virus; MOI, multiplicity of infection; SD, standard deviation; VP40, viral protein 40.

H84T BanLec was prepared in *E*. *coli* as previously described [16], except that non-His-tagged protein was used for the majority of the study (in all experiments except those depicted in Figs 1, 2B and 7) and purified on a Sephadex G-75 column instead of on a Ni-NTA agarose column. Briefly, cleared bacterial lysates were added to the Sephadex column equilibrated with PBS and the column washed with PBS until the OD of the flow-through at 280 nm was < 0.02. The protein was then eluted with 0.2 M methyl-α-D-glucopyranoside. WT and D133G BanLec were prepared as previously described [16] and were His-tagged. For all lectins, endotoxin levels were tested with the Pierce LAL Chromogenic Endotoxin Quantitation Kit (Thermo Fisher Scientific). To remove endotoxin, 1 M glucose was added and pooled eluates containing the protein were passed through Mustang E filters (Pall). Following endotoxin removal to < 0.1 endotoxin units/mg of protein, the Vivaspin 20 centrifugal unit with 3K MWCO was used to remove glucose and concentrate H84T in water. Protein and endotoxin concentrations were then tested using the Pierce BCA Protein Assay Kit (Thermo Fisher Scientific) and LAL assay, respectively.

Ribavirin and toremifene citrate were purchased from Selleck.

### Construction and expression of KZ52-scFv antibody fragments in E. coli

KZ52-scFv, which recognizes EBOV GP, was expressed and purified by the Antibody Engineering and Technology core at the University of Virginia. To do so, a gene fragment consisting of codon-optimized DNA sequences for the KZ52 variable heavy chain linked to the KZ52 variable light chain by a 15 amino acid linker (Gly_4_Ser)_3_ was synthesized by BioBasic. The gene was cloned into a pET-based vector containing a 6x histidine tag and a *pelB* leader sequence for periplasmic expression of protein. *E*. *coli* B strain competent cells engineered to promote disulfide bond formation in the cytoplasm that is suitable for T7 promoter driven protein expression (designated SHuffle T7 Express: *fhuA2 lacZ*:*T7 gene1 [lon] ompT ahpC gal λatt*::*pNEB3-r1-cDsbC (Spec*^*R*^, *lacI*^*q*^*) ΔtrxB sulA11 R(mcr-73*::*miniTn10—Tet*^*S*^*)2 [dcm] R(zgb-210*::*Tn10—Tet*^*S*^*) endA1 Δgor Δ(mcrC-mrr)114*::*IS10*) were transformed with the pET-KZ52 construct and grown on ampicillin-LB agar plates.

The transformed cells were grown using rich medium, 2xYT, with ampicillin, at 37°C for 16 h. The next day, pre-warmed 2xYT medium was prepared in 2 L conical flasks (300 mL per flask) with antibiotics, inoculated with 10 mL of the overnight culture and then grown at 37°C (250 rpm) until the optical density at 600 nm had reached 0.5. Cells were then placed on ice for 30 min and IPTG was added to a final concentration of 0.5 mM. The cultures were grown at 25°C (250 rpm) for an additional 16 h. Bacterial cells were pelleted by centrifugation for 20 min (11,000 x g, 4°C), the supernatants were discarded, and the pellets were frozen at -20°C until needed.

#### Purification of scFv fragments from periplasm

Periplasmic extracts were prepared from bacterial cells as previously described [22]. Briefly, bacterial cell pellets were re-suspended in 1/50 volume of lysis buffer (10 mM HEPES, pH 7.4, 1 mg/mL hen egg lysozyme, and an EDTA-free protease inhibitor cocktail [Halt protease inhibitor cocktail, Thermo Fisher Scientific]). The cells were incubated on ice for 1 h. After incubation, MgCl_2_ and DNase I were added to final concentrations of 10 mM and 20 U/mL, respectively. The cells were incubated at room temperature for 30 min and pelleted by centrifugation at 4°C (20 min, 18,000 x g). The supernatants were collected and stored on ice. Antibody scFv fragments were purified from these periplasmic extracts by immobilized metal affinity chromatography (IMAC). The IMAC resin (His-60, Clontech) was equilibrated in HEPES buffer supplemented with 10 mM imidazole and 300 mM NaCl (pH 7.4). Periplasmic extract from 2 L of bacterial culture was mixed with 3 mL of equilibrated resin and incubated on an end-over-end rotator for 1 h at 4°C. The resin was subsequently packed into a column (0.8 x 4 cm) and washed with 20 bed volumes of HEPES buffer supplemented with 10 mM imidazole and 300 mM NaCl and 5 bed volumes of HEPES supplemented with 20 mM imidazole and 300 mM NaCl. The scFv was eluted from the column using an imidazole step gradient from 100 to 250 mM imidazole in HEPES containing 300 mM NaCl. Protein elution was monitored by UV absorbance at 280 nm. The collected fractions were analyzed by SDS-PAGE.

### Viruses

Ebola virus/H.sapiens-tc/GIN/2014/Makona-C05 (EBOV/Mak, GenBank accession no. KX000398.1) and mouse-adapted Ebola virus/Mayinga (GenBank accession no. KY425637.1) (ma-EBOV) were propagated as previously described [23]. Virus stock and challenge inoculum titers were determined by plaque assay on Vero E6 cells. All procedures using live EBOV were performed under BSL-4 conditions.

### Infection studies

#### In vitro cell-based infection assay

The cell-based EBOV drug screen assay was performed as previously described [24]. Briefly, Huh 7 or Vero E6 cells were seeded at a density of 3 x 10^4^ cells/well in black, clear bottom 96-well plates approximately 24 h before adding H84T BanLec. H84T was diluted in DMEM with 10% HI-FBS and added to the cell media to a final concentration ranging from 0.16 μM to 20 μM. After 1 h, cells were infected with EBOV/Mak at the indicated multiplicity of infection (MOI). After 48 h, plates were fixed with 10% neutral-buffered formalin. EBOV was detected with a mouse antibody against EBOV viral protein 40 (VP40) (#B-MD04-BD07-AE11, made by the United Stated Army Research Institute of Infectious Diseases under Centers for Disease Control and Prevention contract). The VP40 antibody was used at a dilution of 1:2500 (in 3% BSA) for staining with Alexa Fluor 594-labeled IgG-antibody (#A11005, Life Technologies, Grand Island, NY; diluted 1:2500 in 3% BSA) and at a dilution of 1:4000 (in 3% BSA, 2% milk) for staining with the horseradish peroxidase (HRP)-conjugated IgG-antibody (SeraCare, Milford, MA, cat. #074–1802; diluted 1:4000 in 3% BSA, 2% milk). The Pico Chemiluminescent Substrate (Thermo Fisher Scientific, Inc., Rockford, IL) was prepared by mixing Luminol/Enhancer and Stable Peroxide Solutions 1:1 and added at 100 μl/well according to manufacturer’s instructions. Cytotoxicity was measured 48 h after drug addition to non-virus-infected plates (black opaque) using a Cell Titer Glo luminescent cell viability assay kit (Promega, Madison, WI). Fluorescence and luminescence were measured using a Tecan plate reader (Infinite M1000, Tecan US, Morrisville, NC).

For efficacy plates, the average background signal from uninfected control wells was subtracted from the signal obtained for all infected wells on each plate. For cytotoxicity plates, the average background signal from wells containing no cells was subtracted from the signal obtained for all other wells. Infectivity and cell viability were measured as a percentage relative to untreated infected cells and untreated cells, respectively. Percent inhibition was calculated as [1 - (treated virus signal-cell background) / (untreated virus signal-cell background)] x 100. Half maximal inhibitory concentration (IC_50_) was calculated from the non-linear regression analysis fitted curve (log[agonist] vs response [variable slope]) using GraphPad Prism Software (La Jolla, CA). Cytotoxicity was calculated as [1 - (treated cell signal-background) / (untreated cell signal-background)] x 100.

#### Murine Ebola virus infection model

**Fig 3:**
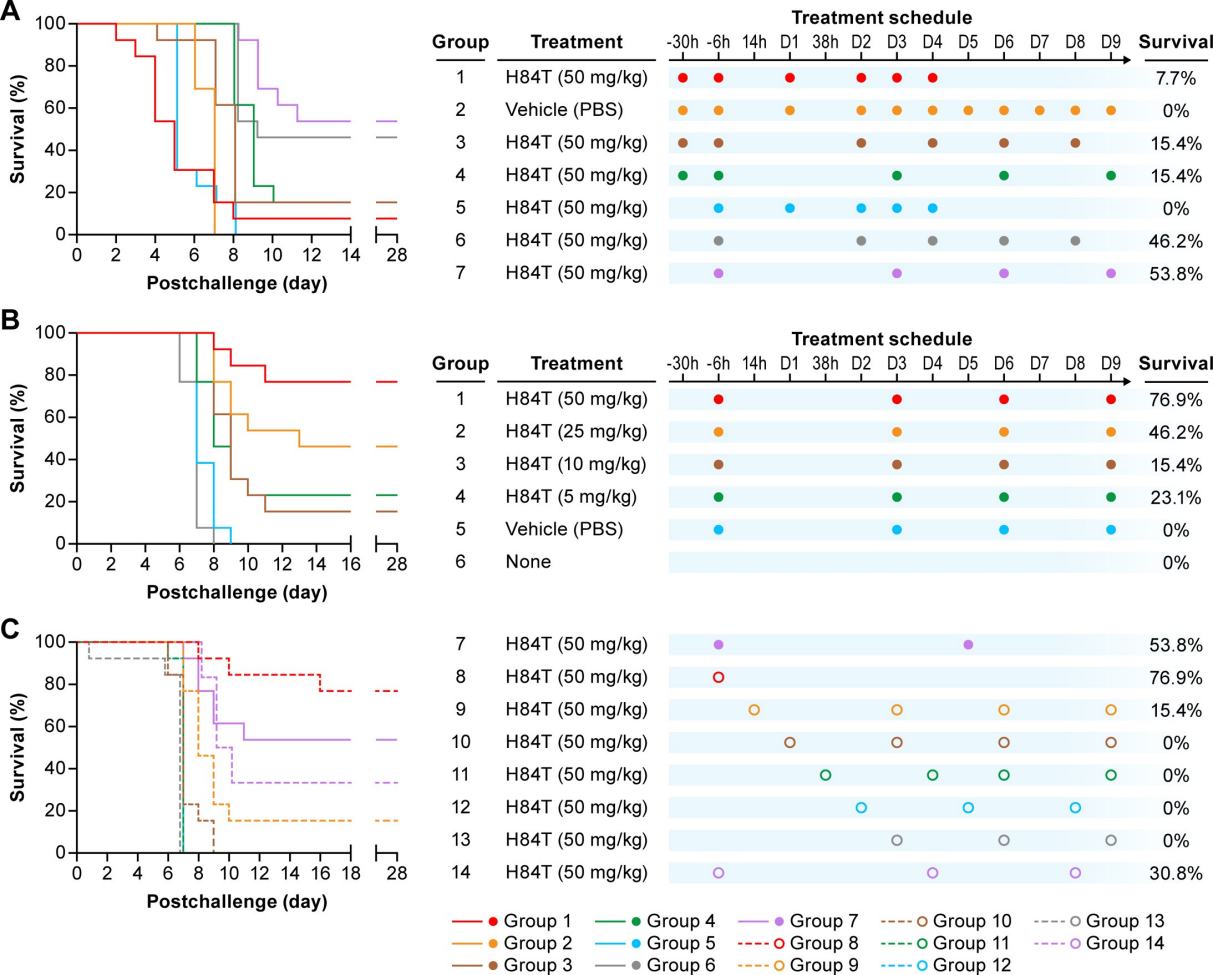
H84T inhibits Ebola virus infection in mice. (A) Survival of mice (n = 13 per group) exposed IP to ma-EBOV (756 PFU) on day 0. H84T treatments were administered IP at 50 mg/kg in PBS at the indicated time points before or after exposure to virus. The control group was treated once daily with vehicle (PBS, Group 2). Treatment for Groups 1 and 5 was discontinued on day 5 due to apparent toxicity. (B, C) Survival of mice (n = 13 per group) exposed IP to ma-EBOV (770 PFU) on day 0. (B) Mice were treated IP with 50, 25, 10, or 5 mg/kg of H84T in PBS at 6 h before exposure to virus and on days 3, 6, and 9 post-exposure. (C) Mice were treated IP with 50 mg/kg H84T in PBS at the indicated time points before or after exposure to virus. Control groups in (B) and (C) received vehicle (PBS, Group 5) or no treatment (None, Group 6). Abbreviations: IP, intraperitoneal; ma-EBOV, mouse-adapted Ebola virus; PFU, plaque forming unit.

Female C57BL/6 mice (Charles River Laboratories, Wilmington, MA) were housed in micro-isolator cages (5 per cage) with CareFresh bedding and provided Teklad autoclavable rodent diet (no. 2018SX, Envigo, Indianapolis, IN) and purified (reverse osmosis) water ad libitum. Groups of mice (n = 13) were challenged intraperitoneally (IP) with a measured dose (calculated by back titration) of 756 and 770 PFU of ma-EBOV on day 0 in the first and second experiments, respectively. Mice were treated IP with H84T BanLec in vehicle (sterile phosphate buffered saline, PBS) starting at 30 h or 6 h before challenge. The treatment doses ranged from 5 to 50 mg/kg and the dosing regimen for each experiment is indicated in Fig 3A, 3B and 3C. Mice were weighed 2–5 days before challenge, and the average mouse weight was used to calculate the treatment dose. A group size of 13 mice was selected to provide 80% power to detect a 0.0 versus 0.5 difference (e.g., 50% survival in an H84T BanLec-treated group compared to 0% survival in the vehicle control group). All personnel participating in the administration of treatment or challenge material, animal assessments, or euthanasia criteria scoring were blinded to the treatment contents (but not dosing schedule) for each group to reduce observer bias.

### Ethics statement

Animals were housed in a facility accredited by the Association for Assessment and Accreditation of Laboratory Animal Care International. All experimental procedures were approved by the National Institute of Allergy and Infectious Diseases, Division of Clinical Research, Animal Care and Use Committee, and were in compliance with the Animal Welfare Act regulations, Public Health Service policy, and the Guide for the Care and Use of Laboratory Animals recommendations.

### trVLP production

trVLPs were prepared under BSL-2 conditions as previously described [25–29]. Briefly, HEK293T/17 cells were seeded in 6-well plates and were transfected at 50–75% confluency with pCAGGS-NP; pCAGGS-VP35; pCAGGS-VP30; pCAGGS-L; a tetracistronic mini-genome plasmid encoding EBOV GP, EBOV VP24, EBOV VP40, and *Renilla* luciferase; and pCAGGS-T7 polymerase using TransIT-LT1 (Mirus). 24 h post transfection, the medium in each well was replaced with 2 mL fresh medium containing 5% serum and 25 mM HEPES (Gibco Life Technologies). 72 h post transfection, the cell medium containing trVLPs was collected, pooled, and cleared of cellular debris by centrifugation (800 x g, 5 min). trVLPs were stored at 4°C until use.

### VLP production

VLPs were prepared as previously described [26,27,30–32]. Briefly, 80% confluent HEK293T/17 cells were transfected with cDNAs encoding EBOV Mayinga GP, VP40, mCherry-VP40, and βlam-VP40. The cell medium was collected 24 and 48 h post transfection, pooled, and cleared of cellular debris by centrifugation (800 x g, 5 min). VLPs in the cleared medium were pelleted through a 20% sucrose cushion by centrifugation (112,398 x g, 2 h) and resuspended in 10% sucrose-HM (20 mM HEPES, 20 mM MES, 130 mM NaCl, pH 7.4). All VLP preps were assessed by western blot analyses and titered on HEK293T/17 cells to confirm entry competency. VLPs were frozen in single use aliquots at -80°C until use.

### trVLP infection (p4cis) assays

Infection of HEK293T/17 cells by trVLPs was assayed as described [25–29]. In short, to prepare target cells, HEK293T/17 cells were seeded in opaque white 96-well plates (Genesee Scientific). The next day, the cells were transfected with pCAGGS-NP, pCAGGS-VP35, pCAGGS-VP30, pCAGGS-L, and pCAGGS-Tim1 using polyethylenimine (PEI, Polysciences Inc). 18–24 h post transfection, target cells were pre-treated for 1 h at 37°C in a 5% CO_2_ incubator with the indicated concentration(s) of the indicated drug(s) diluted in cell medium (medium alone for mock). For some experiments (as noted), the trVLPs (instead of cells) were pretreated in medium containing the indicated concentration(s) of the indicated drug(s) or KZ52-scFv (or medium alone for mock) for 1 h at 37°C.

To assess trVLP infection, the pretreatment solution (or medium above cells for experiments where the trVLPs were pretreated) was removed and replaced with trVLPs diluted in growth medium containing the indicated concentration(s) of the indicated drug(s) or the pretreated trVLPs (as noted). The cells were then incubated for 48 h at 37°C in a 5% CO_2_ incubator, after which time luciferase was measured using Renilla-Glo substrate and a GloMax plate reader (both Promega). Percent inhibition was calculated as [1 - (treated trVLP signal-cell background) / (untreated trVLP signal-cell background)] x 100. Cytotoxicity was calculated as [1 - (treated cell signal-background) / (untreated cell signal-background)] x 100.

### Transcription/Replication (p1cis) assays

HEK293T/17 cells were seeded in 24-well plates. When 50–75% confluent, the cells were pretreated for 1 h at 37°C in a 5% CO_2_ incubator with the indicated concentration(s) of the indicated drug(s) (medium alone for mock). Transfection complexes composed of (per well) 25 ng pCAGGS-NP, 25 ng pCAGGS-VP35, 15 ng pCAGGS-VP30, 200 ng pCAGGS-L, 50 ng of a monocistronic mini-genome plasmid encoding *Renilla* luciferase, 50 ng pCAGGS-T7 polymerase, and 1.5 μL TransIT-LT1 were diluted in Opti-MEM1 (OMEM, Gibco Life Technologies) and added directly to the cells. 24 h post transfection, the medium in each well was replaced with fresh growth medium containing 5% serum, 25 mM HEPES, and the indicated concentration(s) of the indicated drug(s). 48 h post transfection, the medium above the cells was removed and Renilla-Glo was added. Once the cells were lysed (by the Renilla-Glo reagent), lysates were transferred to opaque white 96-well plates (Genesee Scientific) and luminescence was measured by GloMax. Percent inhibition was calculated as [1 - (treated p1cis signal-cell background) / (untreated p1cis signal-cell background)] x 100.

### VLP entry assays

VLP entry assays were performed as previously described [26,27,30–32]. Briefly, HEK293T/17 cells were seeded in 96-well plates coated with fibronectin (Millipore) and were grown overnight at 37°C in a 5% CO_2_ incubator. When the cells were 80–90% confluent, the cells were pretreated with the indicated concentration of H84T Banana Lectin (H84T) diluted in OMEM (water for mock) for 1 h at 37°C in a 5% CO_2_ incubator. For some experiments (as indicated), the VLPs (instead of the cells) were pretreated in OMEM containing the indicated concentration of H84T or KZ52-scFv for 1 h at 37°C. The pretreatment solution (or medium above the cells for experiments where the VLPs were pretreated) was removed and VLPs were bound to the cells by spinfection (250 x g) for 1 h at 4°C. The cells were incubated for 3 h at 37°C in a 5% CO_2_ incubator, the β-lactamase substrate CCF2-AM (Life Technologies) was loaded into cells for 1 h at RT, and the cells were incubated overnight at RT. The cells were then lifted, fixed, and analyzed by flow cytometry as described in detail in Ref. 30. Percent inhibition was calculated as [1 - (treated virus signal-CCF2-AM background) / (untreated virus signal-CCF2-AM background)] x 100.

### VLP assembly and budding assays

HEK293T/17 cells were seeded in 6-well plates. When ~50–75% confluent, the cells were pretreated for 1 h at 37°C in a 5% CO_2_ incubator with the indicated concentration of H84T (water for mock). Transfection mixes comprised of (per well) 0.5 μg EBOV VP40 cDNA, 0.5 μg full-length EBOV Mayinga GP cDNA, 10 μL Lipofectamine 2000 (Invitrogen), and 250 μL OMEM were prepared and added drop-wise to the cells. Following a 24 h incubation at 37°C in a 5% CO_2_ incubator, the supernatant (containing budded VLPs) was harvested and cleared of cellular debris by centrifugation. The VLPs in the clarified medium were pelleted through a 20% sucrose cushion and were resuspended overnight at 4°C in HM buffer. Concurrently, the VLP producer cells were lysed in 1X cold RIPA buffer (150 mM NaCl, 1 M Tris pH 8.0, 1% IGEPAL, 0.1% SDS, 0.5% sodium deoxycholate). The lysates were then cleared of debris by centrifugation and a BCA was performed to determine total protein concentration.

Gels of cell lysates (10 μg) or budded VLPs (from the supernatants) were run, protein was transferred to nitrocellulose, and the membranes were blocked for 1 h at RT. The membranes were then probed overnight for EBOV VP40 and tubulin (loading control) using 1:500 rabbit α-VP40 (IBT Bioservices) and 1:1000 mouse α-tubulin (Sigma) antibodies, followed by washing. EBOV VP40 and tubulin were detected using α-rabbit IR800 and α-mouse IR680 infrared secondary antibodies and an Odyssey CLx infrared imager (all LI-COR Biosciences). Bands corresponding to VP40 and tubulin were quantified using Image Studio Lite (LI-COR Biosciences), and the percentage of VP40 in the supernatant was calculated as the signal intensity of the VP40 band in the supernatant divided by the total signal intensity of the VP40 bands in the supernatant + lysate.

## Results

### H84T inhibits Ebola virus in vitro

In a pilot test, we found that H84T BanLec (H84T) impedes infection by HIV-1 pseudovirions bearing the glycoprotein (GP) from the Mayinga variant of Ebola virus (EBOV). Based on these preliminary findings, we tested the effects of three doses of H84T on the replication of EBOV (Makona variant), the causative agent of the 2013–2016 Western African epidemic of EVD, in Huh 7 cells (derived from human liver) and Vero E6 cells (derived from monkey kidney), cells that are frequently used for in vitro studies of EBOV infection [23–32]. As seen in Fig 1, H84T blocked the replication of Makona EBOV in Huh 7 and Vero E6 cells, yielding 96 and 67% inhibition, respectively, at the highest concentration tested (20 μM). We next performed 8-point dose response studies to further assess the effects of H84T. As seen in Fig 2A, H84T inhibited replication of Makona EBOV by ~50% at the highest concentration (20 μM) tested in both Huh 7 and Vero E6 cells. Before testing H84T in the mouse model, we tested its effects on infections by mouse-adapted EBOV (ma-EBOV), which is derived from Mayinga EBOV [33]. Inhibition of ma-EBOV appeared somewhat stronger, reaching ~70–90% inhibition, with IC_50_ values of ~6 and ~1 μM, respectively, in Huh 7 and Vero E6 cells (Fig 2B).

### H84T inhibits Ebola virus in murine infection models

Given that H84T inhibited the replication of Makona EBOV and ma-EBOV in cell cultures, we tested its ability to block EBOV infection in vivo in a murine model of disease. Initial dosing strategies were designed based on prior experience with efficacy against influenza A virus as well as pharmacodynamic data in mice (E. Covés-Datson and D. Markovitz, manuscript in review). As seen in Fig 3A, in a first study, two H84T dosing regimens (Groups 6 and 7) led to partial (~50%) protection: when H84T was administered at a dose of 50 mg/kg intraperitoneally (IP) 6 h prior to virus exposure and then again either on days 2, 4, 6, and 8 or on days 3, 6, and 9 (i.e. with either one or two days in between doses). No vehicle-treated mice survived. Other dosing regimens (Fig 3A: Groups 1, 3, 4, and 5) yielded less or no protection. In a second study, H84T was administered 6 h prior to exposure and then again on days 3, 6 and 9, with either the prior (50 mg/kg) or lower (5, 10, or 25 mg/kg) doses. A slightly higher level of protection (~77%) was observed at the 50 mg/kg dose (Fig 3B: Group 1) compared to that seen in the first study (~50%) (Fig 3A: Groups 6 and 7). Using this dosing schedule, as the dose of H84T was reduced there was a decrease in the survival benefit (Fig 3B: Groups 2–4). In the second experiment, we also tested the 50 mg/kg dose of H84T when administered in alternative regimens. Interestingly, the same level of protection (~77%) was observed when a single 50 mg/kg dose of H84T was administered 6 h prior to virus exposure (Fig 3C: Group 8) as when four 50 mg/kg doses of H84T were administered, 6 h prior to exposure and then again on days 3, 6, and 9 post-exposure (Fig 3B: Group 1). Alternative regimens that lacked a 50 mg/kg dose of H84T 6 h prior to challenge resulted in no protection with the exception of Group 9 (15.4% protection), which received the first dose at 14 h post-virus exposure (Fig 3C: Groups 9–13). Consistent with the findings in Fig 3A, less frequent dosing with H84T and a dose at 6 h prior to virus exposure resulted in higher levels of protection. Therefore, the findings from the second test of H84T in EBOV-challenged mice (displayed in Fig 3B and 3C) confirmed and extended those from the first test (shown in Fig 3A), demonstrating that a single dose of H84T provides reasonably effective prophylaxis against EVD.

### Mechanism of Ebola virus inhibition by H84T BanLec

**Fig 4:**
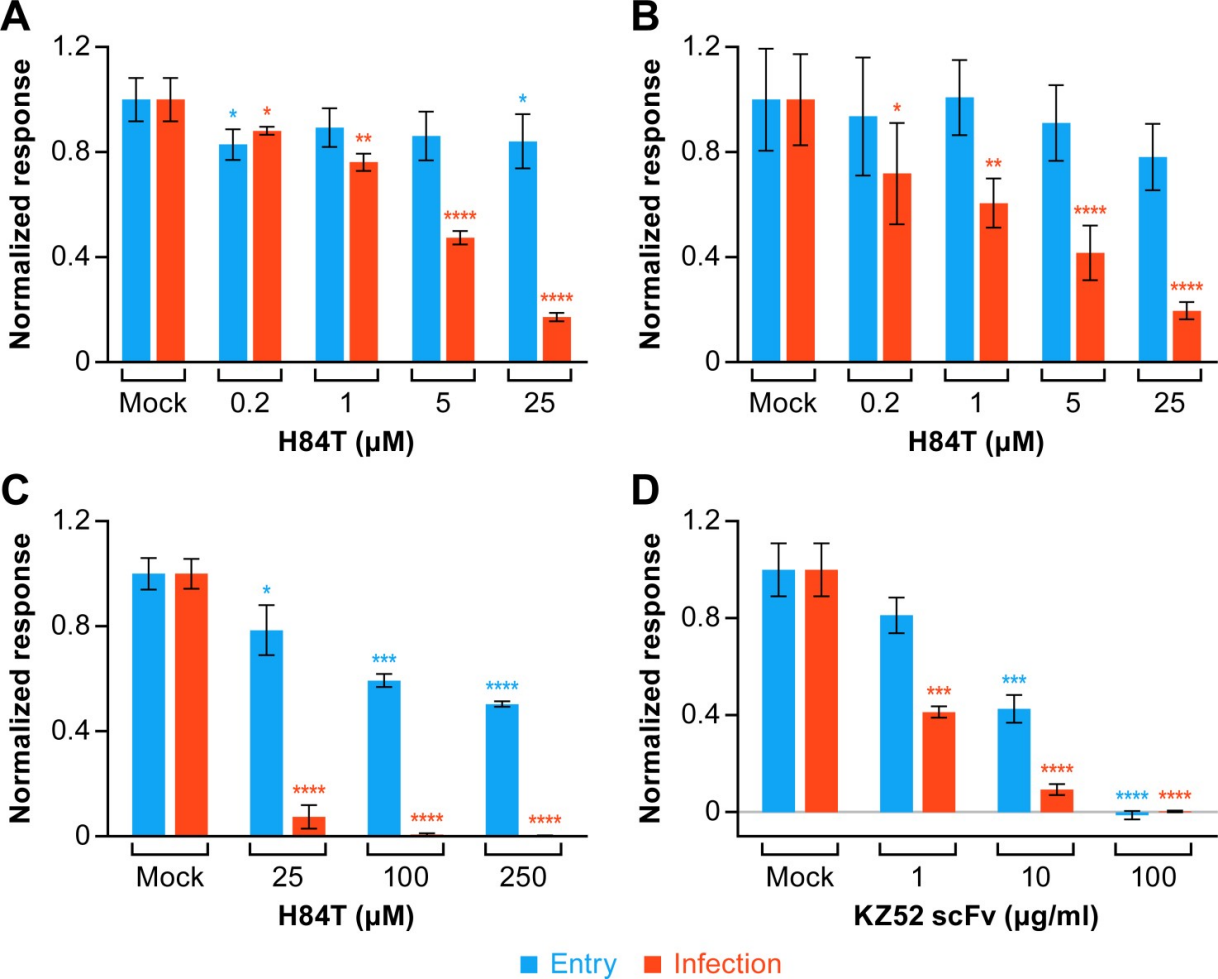
H84T blocks Ebola VLP entry, but less potently than it blocks infection by trVLPs. (A) VLPs and trVLPs or (B,C) 293T/17 cells were pretreated with the indicated concentration of H84T for 1 h at 37°C. Cells were then processed and analyzed for VLP entry and trVLP infection, with H84T present throughout, as described in the Methods section. VLP entry (blue bars) and trVLP infection (red bars) were scored at 3 and 48 h post addition of VLPs or trVLPs, respectively. (D) VLPs and trVLPs were pretreated with the indicated concentration of the anti-EBOV single chain antibody KZ52-scFv for 1 h at 37°C. Cells were then processed and analyzed as in panels A-C. Experiments depicted in panels C and D were performed on the same day with the same set of target cells. Data are averages of triplicate samples. Error bars represent the SD of the triplicates. Asterisks represent: *p<0.05, **p<0.01, ***p<0.001, and ****p<0.0001 based on two-tailed student t-tests.

In the case of HIV-1, wild-type (WT) BanLec binds to high-mannose sugars on the gp120 envelope glycoprotein and blocks HIV-1 infection by inhibiting virus attachment to and fusion with host cells [34]. We therefore tested if H84T might exert its inhibitory effect against EBOV by blocking EBOV entry into host cells. To do this we directly compared the ability of H84T to block infection by transcription- and replication-competent virus-like particles (trVLPs), a surrogate assay for authentic EBOV infection [25–29], and to block entry of EBOV VLPs; both assays can be conducted under BSL-2 conditions. The VLP entry assay employs filamentous entry-reporter VLPs containing the EBOV matrix protein (VP40) and glycoprotein (GP) as well as the entry reporter, VP40 β-lactamase [30]. Entry reporter VLPs enter host cells (monitored by cleavage of a fluorescent β-lactamase substrate), but do not perform further stages of the EBOV life cycle, as they do not contain a viral genome or replication machinery proteins. trVLPs are morphologically similar to entry reporter VLPs and virus (i.e., they are filamentous) and they also contain EBOV VP40 and GP. However, trVLPs additionally contain a 4-cis mini-genome (encoding *Renilla* luciferase, VP40, GP, and VP24, flanked by 5’ leader and 3’ trailer sequences from the EBOV genome) as well as EBOV proteins that drive EBOV replication: NP, L, VP30 and VP35. After trVLPs enter a cell, they replicate the 4-cis mini-genome and transcribe its encoded genes including *Renilla* luciferase, which is easily monitored by a *Renilla* luciferase assay (see Fig 1 in Watt and Hoenen [25]). When trVLPs and VLPs were pre-treated with H84T (and then H84T was maintained in the cultures), the engineered lectin strongly inhibited trVLP infection, with an apparent IC_50_ of ~5 μM, but showed minimal inhibition of VLP entry (Fig 4A). We also tested the effects of pre-treating the target cells with H84T (and then maintaining it throughout the experiment). H84T behaved similarly, showing strong inhibition of trVLP infection but no significant inhibition of VLP entry (Fig 4B). Hence, whether the VLPs/trVLPs or the target cells were pre-treated with up to 25 μM H84T, the engineered lectin did not inhibit the signal in the VLP entry assay but had a strong inhibitory effect on trVLP infection.

Where studied, low molecular weight inhibitors that block Ebola entry by targeting host cell pathways block trVLP infection and VLP entry with similar potencies [26,27]. Expecting H84T to be an EBOV entry inhibitor, we were therefore surprised that it inhibited trVLP infection much more strongly than VLP entry (Fig 4A and 4B). To explore this observation further, we first tested higher concentrations of H84T. As seen in Fig 4C, higher concentrations of H84T (100 and 250 μM) did inhibit VLP entry, but as seen previously (Fig 4A and 4B) considerably less potently than trVLP infection. In parallel with the experiment depicted in Fig 4C, we tested the effects of a single chain form of the KZ52 mAb (i.e., a virus-targeted biological inhibitor) on trVLP infection and VLP entry. As seen in Fig 4D, KZ52-scFv also inhibited trVLP infection more strongly than VLP entry, but the differential effect was not as strong as seen with H84T.

Given that H84T strongly blocked trVLP infection with only relatively weak activity on VLP entry, we tested whether it inhibits post-entry phases of the EBOV life cycle. We first assessed the effects of H84T in an assay that measures genome transcription and replication independently of EBOV entry. To do this we compared the effects of H84T on the *Renilla* luciferase read-outs from ‘1-cis’ (monocistronic) and ‘4-cis’ trVLP assays [25,28]. Unlike the 4-cis assay described above (referred to above as the trVLP infection assay), which evaluates the entire EBOV life cycle (including entry), the 1-cis assay strictly measures mini-genome transcription and replication. For the 1-cis assay, cells are co-transfected with a monocistronic mini-genome containing a *Renilla* luciferase reporter (flanked by the EBOV 5’ leader and 3’ trailer sequences) along with cDNAs encoding T7 polymerase, and the EBOV replication proteins NP, VP30, VP35, and L. Hence, within the transfected cells, the mini-genome is transcribed and *Renilla* luciferase is produced (see Fig 2 in Hoenen et al. [28]).

**Fig 5:**
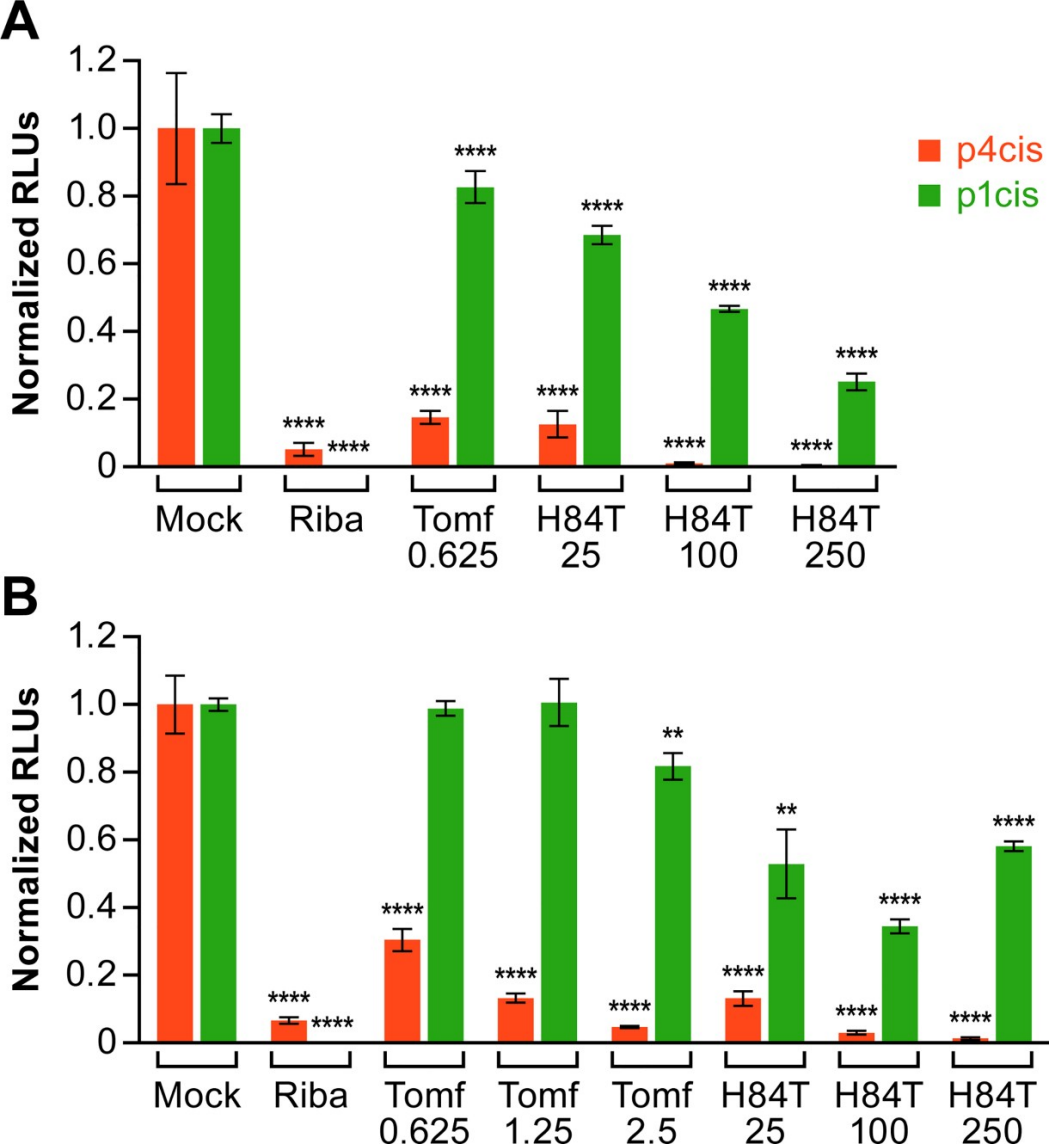
H84T blocks Ebola virus genome transcription/replication, but less potently than it blocks infection by trVLPs. (A) Parallel sets of 293T/17 cells (one set of trVLP target cells and one set of untransfected cells, at 50–75% confluency) were pretreated for 1 h at 37°C with ribavirin (Riba, 50 μg/mL), Toremifene (Tomf, 0.625, μM), or H84T (25, 100, or 250 μM) and maintained in the same concentration of inhibitor for the remainder of the experiment. After the preincubation period, trVLPs were either added to the trVLP target cells for the p4cis “trVLP infection” assay (red bars) or cells were transfected with the p1cis monocistronic mini-genome and associated RNP plasmids, for the p1cis “genome replication” assay (green bars). Luciferase activity was measured 48 h post infection or transfection. Both assays are described in the Methods section. (B) A repeat experiment was conducted as in (A), but with the addition of two higher concentrations of toremifene (1.25 and 2.5 μM). Data are the averages of triplicate samples, and error bars represent SD of the triplicate means. Asterisks indicate (**) p<0.01 and (****) p< 0.0001, based on two-tailed student t-tests, relative to respective mock treated controls.

As expected, ribavirin, a known EBOV replication inhibitor [27], blocked genome replication (1-cis assay) as strongly as it inhibited the trVLP full life cycle (4-cis assay) (Fig 5; panels A and B represent two independent experiments). The entry inhibitor toremifene [29,31,32] blocked the full life cycle assay (4-cis, which requires VLP entry and subsequent steps of the EBOV life cycle), the expected behavior of an entry inhibitor, while having only a small effect (0–18% inhibition) on the 1-cis genome transcription/replication assay. In parallel experiments, H84T exerted a stronger effect (31–76% inhibition) than toremifene on genome transcription/replication (1-cis assay), but the inhibition of transcription/replication caused by H84T was weaker than its corresponding effects on the full (4-cis) life cycle assay.

**Fig 6:**
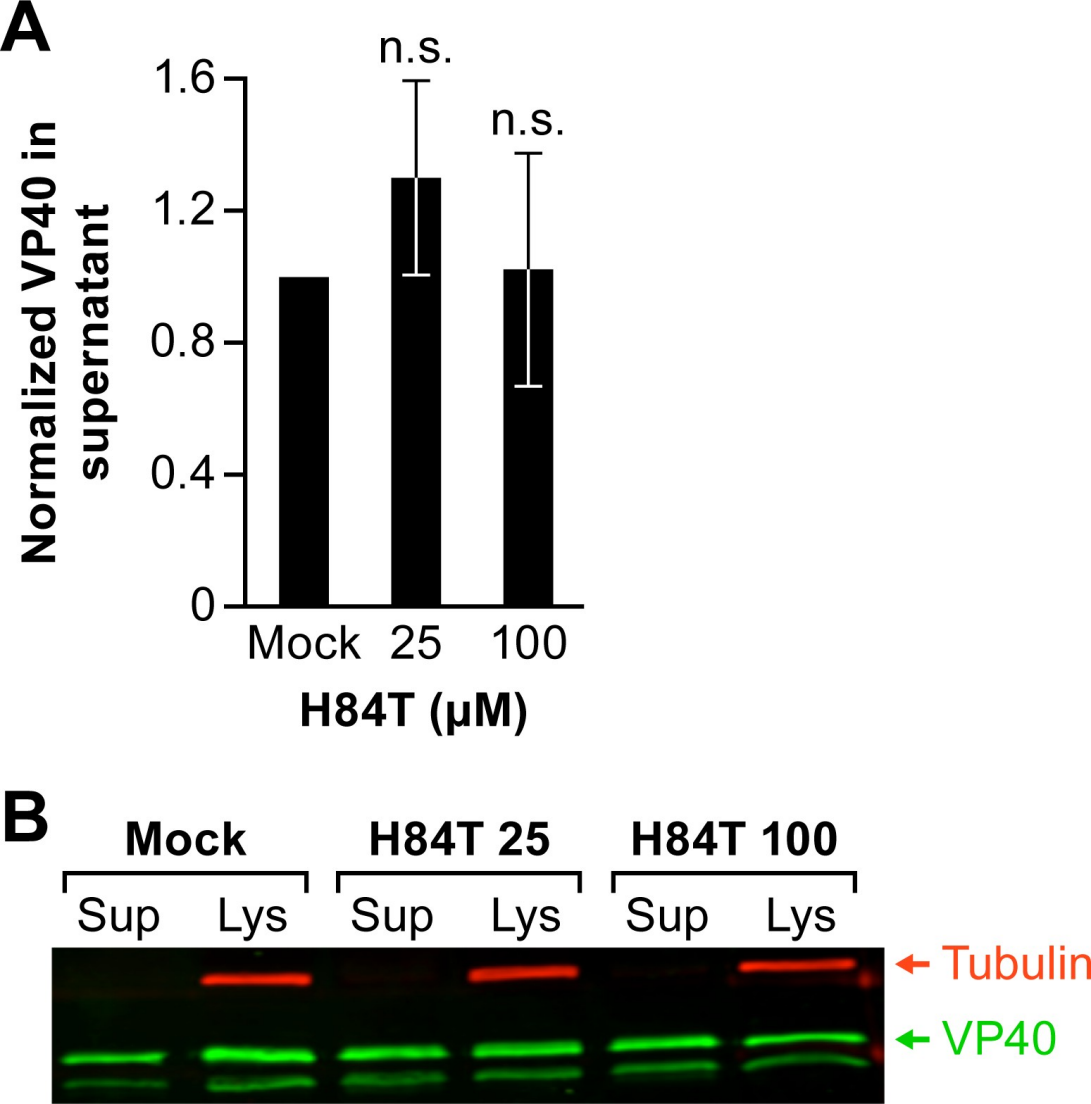
H84T does not block Ebola VLP budding or release. 293T/17 cells were pretreated for 1 h at 37°C with the indicated concentration of H84T and then transfected with plasmids encoding VP40 and Ebola GP. 24 h post transfection (in the continued presence of H84T), particles that had been released into the medium were collected, cleared of cell debris, and concentrated by centrifugation. The cells were then lysed (as described in the Methods section). Samples of cell lysates and released particles were then subjected to SDS-PAGE and analyzed by western blotting for the presence of VP40. Tubulin was used as a loading control for cell lysate samples. The extent of VLP release was calculated as the ratio of the VP40 band intensity in the supernatant divided by the VP40 band intensity in the supernatant + lysate. Data in (A) are the averages (+/- SEM) from 4 independent experiments (one set of samples per experiment). The blot from a representative experiment is shown in (B). ns: not statistically significant (p = 0.27, 25 μM; p = 0.9, 100 μM) based on two-tailed student t-tests.

Since H84T exerts a stronger effect on the trVLP full life cycle assay (requiring entry, replication/transcription, and assembly/budding) with weaker effects in the VLP entry (Fig 4) and transcription/replication (Fig 5) assays, we next asked whether it affects the other major stage of the EBOV life cycle involving new particle assembly, budding, and release from producer cells. To do this, we monitored the effects of H84T on the budding of EBOV VLPs containing EBOV VP40 and EBOV GP (but no other EBOV proteins), using an assay described by Loughran et al. [35]. There was no apparent effect of H84T on the budding of EBOV VLPs (Fig 6), suggesting that H84T does not exert its inhibitory effect against EBOV via inhibition of virus assembly, budding, or release.

**Fig 7:**
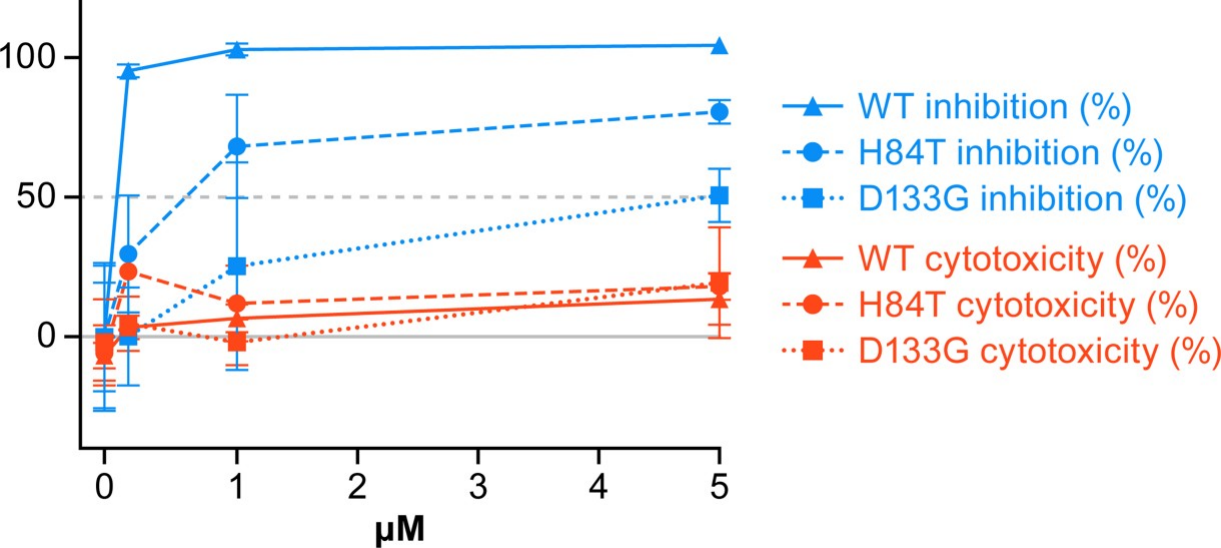
Comparative effects of WT, H84T and glycan binding-deficient D133G BanLec on EBOV trVLP infection and cytotoxicity. 293T/17 cells were pretreated with the indicated concentrations of WT (triangles; solid lines), H84T (circles; dashed lines), or D133G (squares; dotted lines) BanLec for 1 h and then processed for trVLP infection (blue) as in the legend to Fig 4 and for cytotoxicity (red) as described in the Methods section. Data (y-axis) are the averages of triplicate samples +/- SD: % cytotoxicity (red), % inhibition of infection (blue).

We next sought to further investigate the unexpected observation that H84T blocks trVLP infection more strongly than VLP entry, reasoning that inhibition of trVLP infection by H84T could potentially be due, in part, to carbohydrate-independent effects. Thus, we compared the anti-trVLP infection activity of H84T and WT BanLec to that of D133G BanLec (D133G) (Fig 7), a variant of banana lectin that is identical to H84T except that, instead of the H84T mutation, it contains a single amino acid mutation in one of its two carbohydrate binding sites and therefore is not thought to efficiently bind to carbohydrates [16]. Indeed, in accordance with decreased binding to carbohydrates, D133G completely lacks antiviral activity against HCV, HIV-1, and influenza A and B viruses [16; and E. Covés-Datson and D. Markovitz, manuscript in review]. As expected based on findings in Fig 4, we found that H84T strongly inhibits trVLP infection. WT BanLec exhibited somewhat stronger inhibition of trVLP infection, which is consistent with previous in vitro studies comparing the activity of WT and H84T BanLec [16]. Surprisingly, D133G was also able to inhibit trVLP infection, though its inhibitory effect was diminished compared to that of H84T or WT BanLec. Cytotoxicity on 293T cells was minimal for all three lectins tested. The finding that D133G inhibits some trVLP infection was unexpected given that it does not even minimally inhibit HIV-1, HCV, or influenza A or B virus replication at doses at which H84T blocks these viruses. It appears that some carbohydrate binding is required for the inhibitory effect of BanLec against EBOV, as the carbohydrate-binding WT and H84T BanLec block trVLP infection more potently than does D133G BanLec. However, that D133G, a carbohydrate binding site mutant, inhibits trVLP infection suggests that at the concentrations used, BanLec may exert some carbohydrate-independent effects, e.g., against EBOV replication. Alternatively, we cannot rule out that D133G might retain weak binding to carbohydrates, which could account for its corresponding weak inhibition of trVLP infection.

## Discussion

Outbreaks of EVD continue to wreak havoc on a global scale and are capable of causing many thousands of deaths and widespread disruption in the regions where the virus emerges. These outbreaks often also spread panic and fear in other regions over the possibility of imported cases, which occurred during the 2013–2016 Western African Ebola epidemic. At the time of this writing, there is an ongoing Ebola epidemic in the Democratic Republic of the Congo that began in August 2018 and is as of yet uncontrolled, in part due to military conflict in the region; this outbreak follows on the heels of another unrelated outbreak in the same country in May-July 2018. Efforts to control Ebola outbreaks have historically relied on general infection control techniques, since to date there is no licensed vaccine or treatment against ebolaviruses. Several vaccine candidates have demonstrated safety and varying degrees of immunogenicity in people, but thus far only the rVSV-ZEBOV vaccine is being used clinically. On the therapeutic side, although some promising candidates exist, including ZMapp and remdesivir, no anti-ebolavirus treatments have yet been licensed, and there remains a need to develop additional potential anti-ebolavirus therapies, ideally ones in ample supply and not requiring a cold chain.

In this work, we first demonstrated that an engineered banana lectin, H84T BanLec (H84T), is active against EBOV in cell cultures. It inhibits both the Makona strain of EBOV, the strain responsible for the 2013–2016 epidemic, which was the largest in history, and the mouse-adapted Mayinga strain of EBOV in the two cell lines tested.

In a murine model of EBOV, we first found that a 50 mg/kg dose of H84T administered IP 6 h pre-exposure to the virus and then every 2 or 3 days thereafter for 3 to 4 additional doses protected ~50% of mice from EBOV infection. Dosing with 50 mg/kg of H84T more frequently was less efficacious, as when H84T was administered twice before exposure to the virus or every day (for 9 days), mouse survival was significantly reduced. In a second experiment, we found that H84T was most efficacious, protecting 77% of mice from otherwise lethal infection when administered 6 h pre-exposure to the virus and then again on days 3, 6 and 9 post-exposure. Lower doses were less efficacious as compared to the 50 mg/kg dose when administered in the same dosing schedule (i.e., 6 h before virus exposure and then on days 3, 6, and 9). Most interestingly, a single dose administered 6 h pre-exposure to the virus also yielded 77% survival. These latter findings suggest that H84T, in conjunction with vaccination, could have potential as a prophylactic agent for health care workers before beginning to treat EVD patients or for warfighters before going off to war. We have previously found that H84T can be toxic to mice when administered at high concentrations (200 mg/kg) (E. Covés-Datson and D. Markovitz, manuscript in review) which, along with the long half-life of H84T, likely explains why some dosing schedules that included more frequent doses of H84T were not as efficacious in the present study. In any case, it appears to be contraindicated to administer H84T to EBOV-infected mice on consecutive days.

When we examined the mechanism of action of H84T against EBOV, we found that at lower micromolar concentrations H84T inhibits EBOV infection, but not viral entry, as indicated by the fact that H84T robustly inhibited infection by trVLPs while having no or minimal effects on VLP entry. This was surprising since H84T is predicted to bind to EBOV GP, the EBOV entry protein. At very high concentrations, above 25 μM, H84T was able to inhibit entry of EBOV VLPs, but not as efficiently as it inhibited trVLP infection, which measures the full viral life cycle and was inhibited at much lower concentrations of H84T. The same higher concentrations of H84T blocked transcription and/or replication of the mini-genome (i.e., in the 1-cis assay), but, as seen for VLP entry, not as potently as they inhibited trVLP infection, suggesting that inhibition of the full life cycle was stronger than inhibition of EBOV genome transcription/replication alone. However, even high concentrations of H84T did not block VLP budding or release. Taken together, these findings indicate that H84T may inhibit EBOV infection by blocking both viral entry and transcription and/or replication. The observed post-entry effects may be due, in part, to non-carbohydrate binding activities of BanLec, as seen with the D133G mutant. Moreover, given that the inhibitory effect of H84T was considerably more potent for the overall life cycle than for either viral entry or genome transcription and/or replication, it is possible that these anti-EBOV effects are synergistic.

That H84T has demonstrated broad-spectrum activity against many different strains of HIV-1 and 2, HCV, and influenza A and B raises the possibility that H84T may also be broad-spectrum against the different species of pathogenic *Ebolavirus*, all of which possess GP molecules that express high-mannose [18] and so would be potential targets for binding by H84T. Most of the vaccines and several therapeutics in development, including ZMapp, are specific for one particular species of ebolavirus [36]. Thus, these agents currently in development would not likely be suitable for broad-spectrum empiric management of suspected EVD, such as would be required for immediate use in the event of a filovirus outbreak before identification of the virus had occurred. Although activity against only one ebolavirus was tested in the present study, if H84T indeed exhibits activity against additional species of *Ebolavirus*, it could be particularly useful as such an empiric treatment.

Our data demonstrate that H84T may have potential in people as an anti-ebolavirus prophylactic, perhaps in conjunction with vaccination as, remarkably, a single dose of H84T partially protects mice from otherwise lethal EBOV infection and as H84T has a long serum half-life (E. Covés-Datson and D. Markovitz, manuscript in review). H84T could also potentially be used therapeutically, perhaps especially when combined with other anti-ebolavirus agents, which if synergistic, could lower the amount of H84T needed. Remaining questions include why H84T is more potent against other viruses than it is against EBOV and what its precise mechanism of action is on a molecular scale. Whereas the IC_50_ concentration of H84T against EBOV appears to be in the low micromolar range, H84T inhibits HIV-1 and -2, HCV, and some subtypes of influenza A with concentrations in the low nanomolar range [16]. The higher concentration required to inhibit Ebola virus could potentially be attributed to the fact that ebolaviruses possess a very high number and density of glycans on their surfaces as compared to other viruses against which H84T has stronger activity [37–39]. In addition, ebolavirus GPs are heavily *O*-glycosylated [18], in contrast to the glycoproteins of HIV-1 and influenza A, e.g., which contain only *N*-glycans [38,40], and H84T generally does not recognize *O*-glycans [16]. If the mechanism of action of H84T requires its carbohydrate-binding activity, which appears to largely but not entirely be the case, then it is reasonable to hypothesize that a larger number and density of glycans on the surface of EBOV would require a higher concentration of H84T to bind to the glycans, though of course this would need to be tested. An additional, non-mutually exclusive possibility is that it is more difficult to block EBOV entry because EBOV can use TIM and TAM family members [41] in addition to lectins to gain entry into cells.

It is exciting to consider that H84T may inhibit both viral entry and genome transcription and/or replication, as this represents a distinct mechanism of action compared to that of WT BanLec against HIV-1 (primarily attachment inhibition) [34] and to that of H84T against influenza A virus (primarily fusion inhibition; E. Covés-Datson and D. Markovitz, manuscript in review). We hypothesize that the anti-viral entry effect may rely on binding to GP, but exactly how a high-mannose-binding lectin inhibits genome transcription/replication is an open question. It is clear, however, that H84T holds promise as a potential anti-ebolavirus prophylactic and/or therapeutic agent since it inhibits two variants of EBOV in cell cultures, protects mice from EBOV infection, may have a mechanism of action targeting both entry and transcription/replication, and is an inhibitor that could potentially be used in combination with other anti-ebolavirus agents that work through different mechanisms [29].

## References

[1] World Health Organization. Ebola virus disease [Internet]. 2019 [cited 26 May 2019]. Available: https://www.who.int/news-room/fact-sheets/detail/ebola-virus-disease

[2] Bell BP, Damon IK, Jernigan DB, Kenyon TA, Nichol ST, O’Connor JP, et al Overview, Control Strategies, and Lessons Learned in the CDC Response to the 2014–2016 Ebola Epidemic. MMWR Suppl. 2016;65: 4–11. 10.15585/mmwr.su6503a2 27389903

[3] Kennedy SB, Bolay F, Kieh M, Grandits G, Badio M, Ballou R, et al Phase 2 Placebo-Controlled Trial of Two Vaccines to Prevent Ebola in Liberia. N Engl J Med. 2017;377: 1438–1447. 10.1056/NEJMoa1614067 29020589 PMC5705229

[4] Milligan ID, Gibani MM, Sewell R, Clutterbuck EA, Campbell D, Plested E, et al Safety and Immunogenicity of Novel Adenovirus Type 26- and Modified Vaccinia Ankara-Vectored Ebola Vaccines: A Randomized Clinical Trial. JAMA. 2016;315: 1610–23. 10.1001/jama.2016.4218 27092831

[5] Henao-Restrepo AM, Camacho A, Longini IM, Watson CH, Edmunds WJ, Egger M, et al Efficacy and effectiveness of an rVSV-vectored vaccine in preventing Ebola virus disease: final results from the Guinea ring vaccination, open-label, cluster-randomised trial (Ebola Ça Suffit!). Lancet. 2017;389: 505–518. 10.1016/S0140-6736(16)32621-6 28017403 PMC5364328

[6] Medaglini D, Santoro F, Siegrist C-A. Correlates of vaccine-induced protective immunity against Ebola virus disease. Semin Immunol. 2018; 10.1016/j.smim.2018.07.003 30041831

[7] Metzger WG, Vivas-Martínez S. Questionable efficacy of the rVSV-ZEBOV Ebola vaccine. Lancet. 2018;391: 1021 10.1016/S0140-6736(18)30560-9 29565013

[8] National Academies of Sciences E and M. Integrating Clinical Research into Epidemic Response [Internet]. Keusch G, McAdam K, Cuff P, Mancher M, Busta ER, editors. Washington, D.C.: National Academies Press; 2017 10.17226/24739 28696651

[9] Check Hayden E. Experimental drugs poised for use in Ebola outbreak. Nature. 2018;557: 475–476. 10.1038/d41586-018-05205-x 29789732

[10] Qiu X, Wong G, Audet J, Bello A, Fernando L, Alimonti JB, et al Reversion of advanced Ebola virus disease in nonhuman primates with ZMapp. Nature. 2014;514: 47–53. 10.1038/nature13777 25171469 PMC4214273

[11] Sissoko D, Laouenan C, Folkesson E, M’Lebing A-B, Beavogui A-H, Baize S, et al Experimental Treatment with Favipiravir for Ebola Virus Disease (the JIKI Trial): A Historically Controlled, Single-Arm Proof-of-Concept Trial in Guinea. Lipsitch M, editor. PLOS Med. 2016;13: e1001967 10.1371/journal.pmed.1001967 26930627 PMC4773183

[12] Warren TK, Jordan R, Lo MK, Ray AS, Mackman RL, Soloveva V, et al Therapeutic efficacy of the small molecule GS-5734 against Ebola virus in rhesus monkeys. Nature. 2016;531: 381–385. 10.1038/nature17180 26934220 PMC5551389

[13] Hayden FG, Friede M, Bausch DG. Experimental Therapies for Ebola Virus Disease: What Have We Learned? J Infect Dis. 2017;215: jiw496 10.1093/infdis/jiw496 28073859 PMC5853886

[14] Balzarini J. Targeting the glycans of glycoproteins: a novel paradigm for antiviral therapy. Nat Rev Microbiol. 2007;5: 583–597. 10.1038/nrmicro1707 17632570 PMC7098186

[15] Tao S-C, Li Y, Zhou J, Qian J, Schnaar RL, Zhang Y, et al Lectin microarrays identify cell-specific and functionally significant cell surface glycan markers. Glycobiology. 2008;18: 761–9. 10.1093/glycob/cwn063 18625848 PMC2733773

[16] Swanson MD, Boudreaux DM, Salmon L, Chugh J, Winter HC, Meagher JL, et al Engineering a Therapeutic Lectin by Uncoupling Mitogenicity from Antiviral Activity. Cell. 2015;163: 746–758. 10.1016/j.cell.2015.09.056 26496612 PMC4641746

[17] Barrientos LG, O’Keefe BR, Bray M, Sanchez A, Gronenborn AM, Boyd MR. Cyanovirin-N binds to the viral surface glycoprotein, GP1,2 and inhibits infectivity of Ebola virus. Antiviral Res. 2003;58: 47–56. Available: http://www.ncbi.nlm.nih.gov/pubmed/12719006 12719006 10.1016/s0166-3542(02)00183-3

[18] Collar AL, Clarke EC, Anaya E, Merrill D, Yarborough S, Anthony SM, et al Comparison of N—and O -linked glycosylation patterns of ebolavirus glycoproteins. Virology. 2017;502: 39–47. 10.1016/j.virol.2016.12.010 27984785

[19] Feldmann H, Nichol ST, Klenk HD, Peters CJ, Sanchez A. Characterization of filoviruses based on differences in structure and antigenicity of the virion glycoprotein. Virology. 1994;199: 469–73. 10.1006/viro.1994.1147 8122375

[20] Weissenhorn W, Dessen A, Calder LJ, Harrison SC, Skehel JJ, Wiley DC. Structural basis for membrane fusion by enveloped viruses. Mol Membr Biol. Taylor & Francis; 1999;16: 3–9. 10.1080/096876899294706 10332732

[21] Michelow IC, Lear C, Scully C, Prugar LI, Longley CB, Yantosca LM, et al. High-Dose Mannose-Binding Lectin Therapy for Ebola Virus Infection. 10.1093/infdis/jiq025 PMC3071052 21288816

[22] Sun Y, Ban B, Bradbury A, Ansari GAS, Blake DA. Combining Yeast Display and Competitive FACS to Select Rare Hapten-Specific Clones from Recombinant Antibody Libraries. Anal Chem. 2016;88: 9181–9. 10.1021/acs.analchem.6b02334 27571429 PMC5032104

[23] Cong Y, Dyall J, Hart BJ, DeWald LE, Johnson JC, Postnikova E, et al Evaluation of the Activity of Lamivudine and Zidovudine against Ebola Virus. Schindler M, editor. PLoS One. 2016;11: e0166318 10.1371/journal.pone.0166318 27902714 PMC5130197

[24] Postnikova E, Cong Y, DeWald LE, Dyall J, Yu S, Hart BJ, et al Testing therapeutics in cell-based assays: Factors that influence the apparent potency of drugs. Freiberg AN, editor. PLoS One. 2018;13: e0194880 10.1371/journal.pone.0194880 29566079 PMC5864066

[25] Watt A, Moukambi F, Banadyga L, Groseth A, Callison J, Herwig A, et al A novel life cycle modeling system for Ebola virus shows a genome length-dependent role of VP24 in virus infectivity. J Virol. 2014;88: 10511–24. 10.1128/JVI.01272-14 24965473 PMC4178905

[26] Nelson EA, Barnes AB, Wiehle RD, Fontenot GK, Hoenen T, White JM. Clomiphene and Its Isomers Block Ebola Virus Particle Entry and Infection with Similar Potency: Potential Therapeutic Implications. Viruses. 2016;8: 206 10.3390/v8080206 27490565 PMC4997570

[27] Nelson EA, Dyall J, Hoenen T, Barnes AB, Zhou H, Liang JY, et al The phosphatidylinositol-3-phosphate 5-kinase inhibitor apilimod blocks filoviral entry and infection. Kashanchi F, editor. PLoS Negl Trop Dis. 2017;11: e0005540 10.1371/journal.pntd.0005540 28403145 PMC5402990

[28] Hoenen T, Watt A, Mora A, Feldmann H. Modeling the lifecycle of Ebola virus under biosafety level 2 conditions with virus-like particles containing tetracistronic minigenomes. J Vis Exp. 2014; 52381. 10.3791/52381 25285674 PMC4828136

[29] Dyall J, Nelson EA, DeWald LE, Guha R, Hart BJ, Zhou H, et al Identification of Combinations of Approved Drugs With Synergistic Activity Against Ebola Virus in Cell Cultures. J Infect Dis. 2018; 10.1093/infdis/jiy304 29939303 PMC6249579

[30] Shoemaker CJ, Schornberg KL, Delos SE, Scully C, Pajouhesh H, Olinger GG, et al Multiple cationic amphiphiles induce a Niemann-Pick C phenotype and inhibit Ebola virus entry and infection. Rong L, editor. PLoS One. 2013;8: e56265 10.1371/journal.pone.0056265 23441171 PMC3575416

[31] Johansen LM, Brannan JM, Delos SE, Shoemaker CJ, Stossel A, Lear C, et al FDA-Approved Selective Estrogen Receptor Modulators Inhibit Ebola Virus Infection. Sci Transl Med. 2013;5: 190ra79–190ra79. 10.1126/scitranslmed.3005471 23785035 PMC3955358

[32] Johansen LM, DeWald LE, Shoemaker CJ, Hoffstrom BG, Lear-Rooney CM, Stossel A, et al A screen of approved drugs and molecular probes identifies therapeutics with anti-Ebola virus activity. Sci Transl Med. 2015;7: 290ra89 10.1126/scitranslmed.aaa5597 26041706

[33] Bray M, Davis K, Geisbert T, Schmaljohn C, Huggins J. A Mouse Model for Evaluation of Prophylaxis and Therapy of Ebola Hemorrhagic Fever. J Infect Dis. 1999;179: S248–S258. 10.1086/514292 9988191

[34] Swanson MD, Winter HC, Goldstein IJ, Markovitz DM. A lectin isolated from bananas is a potent inhibitor of HIV replication. J Biol Chem. 2010;285: 8646–55. 10.1074/jbc.M109.034926 20080975 PMC2838287

[35] Loughran HM, Han Z, Wrobel JE, Decker SE, Ruthel G, Freedman BD, et al Quinoxaline-based inhibitors of Ebola and Marburg VP40 egress. Bioorg Med Chem Lett. 2016;26: 3429–35. 10.1016/j.bmcl.2016.06.053 27377328 PMC4955528

[36] Lévy Y, Lane C, Piot P, Beavogui AH, Kieh M, Leigh B, et al Prevention of Ebola virus disease through vaccination: where we are in 2018. Lancet (London, England). Elsevier; 2018;392: 787–790. 10.1016/S0140-6736(18)31710-0 30104048 PMC6128979

[37] Francica JR, Varela-Rohena A, Medvec A, Plesa G, Riley JL, Bates P. Steric Shielding of Surface Epitopes and Impaired Immune Recognition Induced by the Ebola Virus Glycoprotein. Basler CF, editor. PLoS Pathog. Public Library of Science; 2010;6: e1001098 10.1371/journal.ppat.1001098 20844579 PMC2936550

[38] Tate MD, Job ER, Deng Y-M, Gunalan V, Maurer-Stroh S, Reading PC. Playing hide and seek: how glycosylation of the influenza virus hemagglutinin can modulate the immune response to infection. Viruses. Multidisciplinary Digital Publishing Institute (MDPI); 2014;6: 1294–316. 10.3390/v6031294 24638204 PMC3970151

[39] Tran EEH, Nelson EA, Bonagiri P, Simmons JA, Shoemaker CJ, Schmaljohn CS, et al Mapping of Ebolavirus Neutralization by Monoclonal Antibodies in the ZMapp Cocktail Using Cryo-Electron Tomography and Studies of Cellular Entry. Sundquist WI, editor. J Virol. 2016;90: 7618–27. 10.1128/JVI.00406-16 PMC4988163 27279622

[40] Stansell E, Panico M, Canis K, Pang P-C, Bouché L, Binet D, et al Gp120 on HIV-1 Virions Lacks O-Linked Carbohydrate. PLoS One. Public Library of Science; 2015;10: e0124784 10.1371/journal.pone.0124784 25915761 PMC4410959

[41] Moller-Tank S, Maury W. Ebola Virus Entry: A Curious and Complex Series of Events. Dutch RE, editor. PLOS Pathog. 2015;11: e1004731 10.1371/journal.ppat.1004731 25928849 PMC4415789

